# Statistically enriched geospatial datasets of Brazilian municipalities for data-driven modeling

**DOI:** 10.1038/s41597-022-01581-2

**Published:** 2022-08-10

**Authors:** Livia Abdalla, Douglas A. Augusto, Marcia Chame, Amanda S. Dufek, Leonardo Oliveira, Eduardo Krempser

**Affiliations:** 1 Oswaldo Cruz Foundation (Fiocruz), Rio de Janeiro, Brazil; 2 Military Institute of Engineering (IME), Rio de Janeiro, Brazil; 3 National Laboratory for Scientific Computing (LNCC), Petrópolis, Brazil

**Keywords:** Environmental sciences, Ecology, Climate sciences, Risk factors

## Abstract

The lack of georeferencing in geospatial datasets hinders the accomplishment of scientific studies that rely on accurate data. This is particularly concerning in the field of health sciences, where georeferenced data could lead to scientific results of great relevance to society. The Brazilian health systems, especially those for Notifiable Diseases, in practice do not register georeferenced data; instead, the records indicate merely the municipality in which the event occurred. Typically in data-driven modeling, accurate disease prediction models based on occurrence requires socioenvironmental characteristics of the exact location of each event, which is often unavailable. To enrich the expressiveness of data-driven models when the municipality of the event is the best available information, we produced datasets with statistical characterization of all 5,570 Brazilian municipalities in 642 layers of thematic data that represent the natural and artificial characteristics of the municipalities’ landscapes over time. This resulted in a collection of datasets comprising a total of 11,556 descriptive statistics attributes for each municipality.

## Abstract (summary)

**Table:** 

Measurement(s)	Socioenvironmental descriptive statistics
Technology Type(s)	PostGIS • QGIS
Sample Characteristic - Environment	All biomes
Sample Characteristic - Location	Brazil

## Background & Summary

The lack of georeferencing of scientific data is a serious problem in various biological, ecological, and epidemiological databases, especially in large countries with high biodiversity, lacking resources for personnel and infrastructure. Spatial referencing of these data is often associated with the names of localities, neighborhoods, municipalities or states, thus conditioning the scale of analysis to regions with known geographic boundaries^1^.

The health and species data without georeferencing restrict the accuracy of spatial analysis in various scientific studies, such as in species distribution modeling^2^ and modeling of emerging zoonoses^3^. Therefore, it will further limit the contribution of the definition of actions and identification of priority areas (states/municipalities) for surveillance, allocation of resources and organization of human care, especially when it comes to diseases with a wide geographic distribution.

In Brazil, the national official public data on diseases and health-related issues from the Ministry of Health are recorded at the municipal level, despite the fact that municipalities present large internal variation in terms of area, population, socioenvironmental and economics characteristics. Of course, the environmental and social processes that either favor or inhibit health risks are not limited to (artificially delimited) administrative divisions^4,5^. Although some Brazilian municipalities have greater detail in the localization of these data, which can be associated with the names of localities or neighborhoods or even georeferenced, this information is nationally scarce and not made publicly available or easily accessible^6^.

According to Barcellos *et al*.^6^, in order for health datasets to serve the purpose of analysis, they need to be georeferenced, integrated with environmental and socioeconomic data, and submitted to procedures for assessment of their spatial distribution. Other studies have also highlighted that knowledge of the environmental, social, and economic characteristics of the location of occurrence of a given epidemiological event is essential for understanding it, since the mere identification of presence or absence of relevant species (hosts, vectors, and etiological agents) and the occurrence of diseases in humans do not suffice to completely understand the spatial distribution pattern of complex and dynamic epidemiological events^7,8^.

Since it is not possible or feasible to retrieve the exact occurrence location of a given epidemiological event, we propose with our datasets an approach to enrich each municipality’s socioenvironmental information, aiming at the improvement of spatial modeling that rely on such spatial aggregation units. For that, we have calculated descriptive statistics for hundreds of thematic layers that cover various natural and artificial features of the landscape over time, leading to many thousands of socioenvironmental statistics for each Brazilian municipality.

It is worth noticing that the statistical characterization is not a substitute for georeferencing; however, by augmenting the information about the municipality, the likelihood of emerging a set of descriptive statistics associated with the underlying phenomenon also increases. Moreover, the methodology adopted here is totally applicable to other spatial reference units (localities, neighborhoods, states). It is useful even when there is georeferencing in order to characterize species’ areas of circulation (buffers).

The application of data-driven modeling methods over the proposed datasets may reveal many indirect or non-obvious relations–but which significantly influence the species and disease distributions–and can contribute to a more in-depth knowledge and clarification of many questions, relations or assumptions that are still not fully understood in Brazil’s complex socioenvironmental context (continental dimension, enormous environmental and ecological diversity, social inequalities, and intense anthropization).

The provided datasets were conceived by our research group in the context of predictive modeling of occurrence of Sylvatic Yellow Fever in Brazil. They were further developed in Abdalla’s 2019 doctoral dissertation^9^ and later they were used to build predictive models which appeared in the “Contingency plan for public health emergency response” published in 2021 by the Ministry of Health^10^.

We believe that our descriptive statistical characterization of the Brazilian municipalities will eventually contribute to society through the advancement of scientific studies that investigate the association of socioenvironmental features with a given event. Furthermore, the methodology employed here can be readily replicated to other countries or regions and thus benefit them as well.

## Methods

The procedure began by obtaining the boundaries of Brazil’s municipalities, which are the most precise spatial reference units available from the Brazilian Ministry of Health of data records on diseases and health events. The boundaries were obtained from the geographic database of the Brazilian Institute of Geography and Statistics (IBGE)^11^, corresponding to the territorial grid of 2015, with a total of 5,570 Brazilian municipalities.

A broad and diverse set of thematic data was used to compose the datasets, spanning a range of time periods (from 1981 to 2021) according to the temporal regularity of individual layers (annual, quinquennial, atemporal, or without temporal regularity), thus covering spatial and temporal variations over Brazil’s territory. It is worth noticing that during the period of 1981 to 2021 the number of municipalities grew from 3991 to 5570^12^, which of course led to major changes to their boundaries, in addition to the creation of the state of Tocantins in 1988 as a result of the division of the state of Goiás^13^. Most of the changes, though, are subdivisions of one municipality into two or more municipalities. To provide statistics that are invariant over the period we would have to resort to using clusters of municipalities (“artificial municipalities”) by means of the Minimum Comparable Areas (MCA) strategy^14^. Due to the time-consuming process we preferred to characterize only the current territorial division, thus providing the most refined statistical characterization of Brazil’s municipalities. Still, one can find it useful to aggregate our characterization according to an MCA territorial division; for that we refer the reader to the article by Ehrl^14^.

**Fig. 1:**
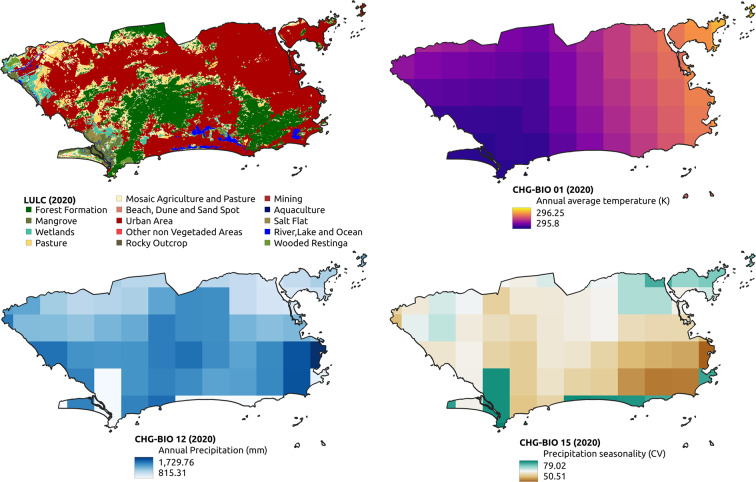
Examples of thematic layers with annual temporality in the territorial extension of the municipality of Rio de Janeiro.

**Fig. 2:**
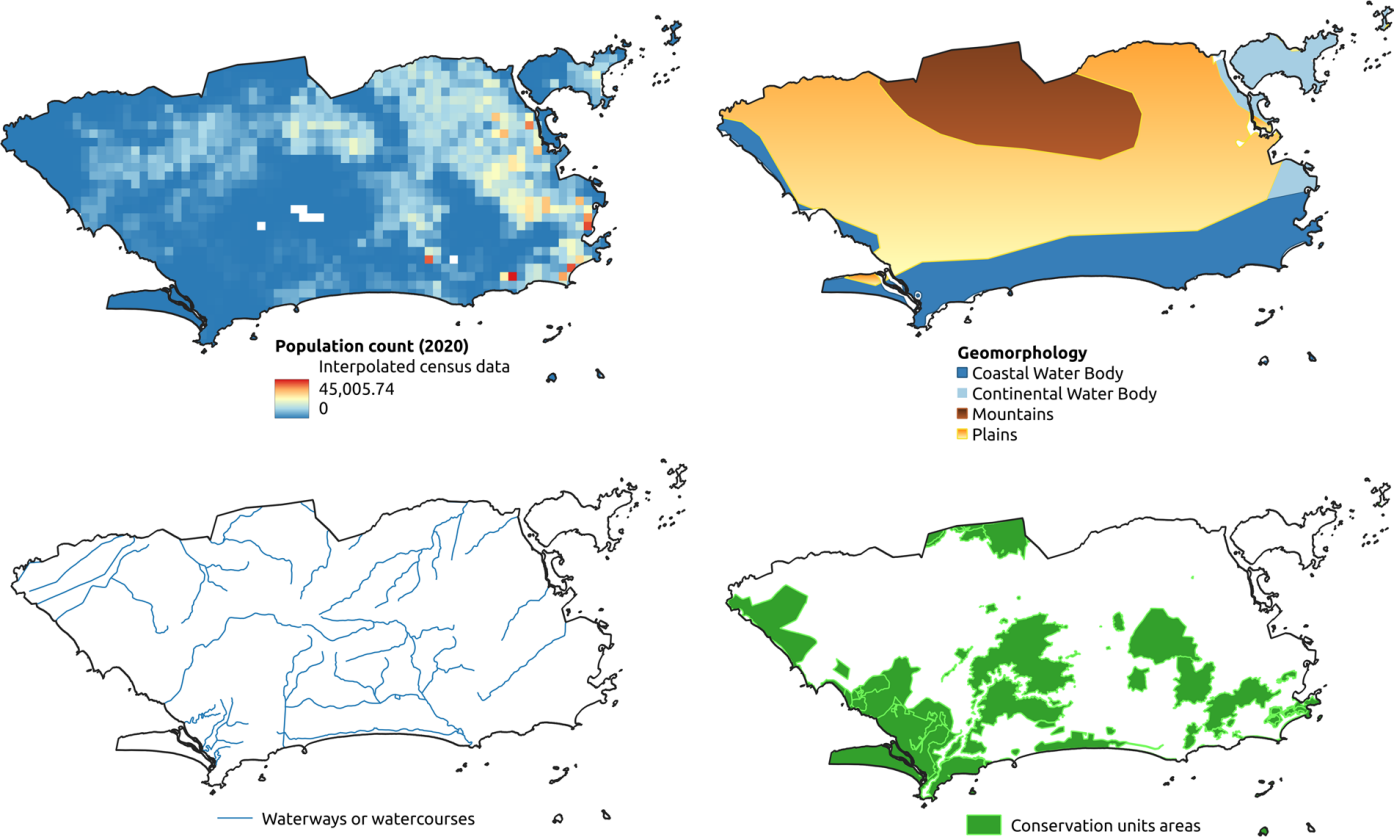
Examples of atemporal and no temporal regularity thematic layers in the territorial extension of the municipality of Rio de Janeiro.

**Fig. 3:**
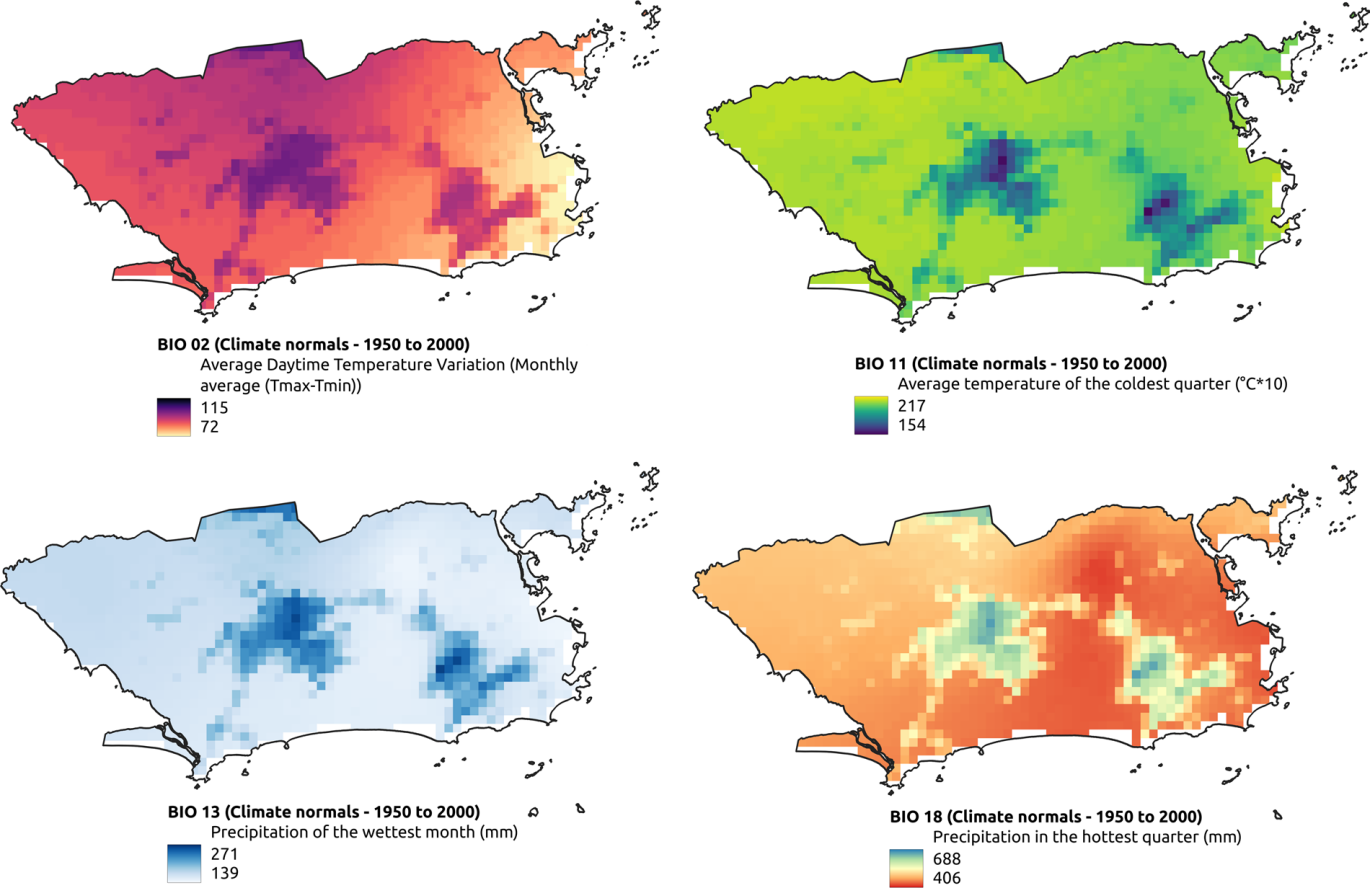
Examples of bioclimatic variables from Worldclim in the territorial extension of the municipality of Rio de Janeiro.

**Fig. 4:**
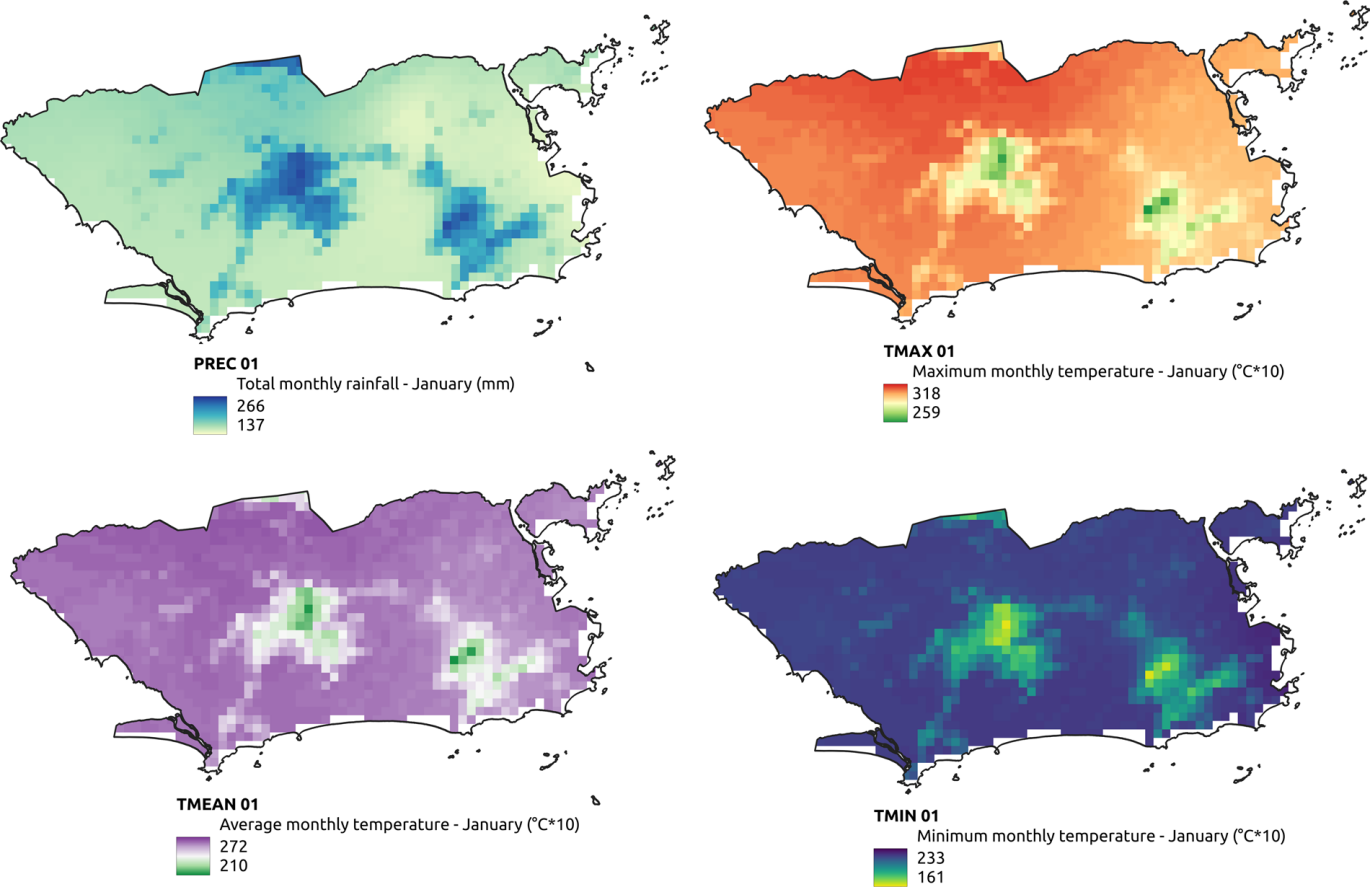
Climate data for total precipitation, maximum, mean and minimum temperature from Worldclim in the territorial extension of the municipality of Rio de Janeiro for the month of January.

A total of 19 thematic layers were used, obtained from different Brazilian government and international agencies (Tables 1 and 2, illustrated by Figs. 1–4). Each layer may have multiple thematic classes or variables, depending on the nature of the theme, totaling 642 thematic classes or variables. For each class, 18 descriptive statistics were calculated (9 raw statistics plus 9 normalized by municipality’s area–Table 3) for all the available years, totaling 11,556 attributes per municipality.

**Table 1:** Thematic layers comprising the dataset collection.

Temporality	Thematic layers	# classes	# attributes	# instances
Annual (1981 to 2020)	Land use and cover (Mapbiomas v6.0; 1985–2020)	25	450	200,484
	Temperature and precipitation (NCEP/CFSR and CHIRPS)	19	198 + 144	445,520
Quinquennial (2000 to 2020)	Population count (SEDAC - NASA)	1	18	27,850
	Population density (SEDAC - NASA)	1	18	27,850
Atemporal	Climate normals for temperature and precipitation (Worldclim)	67	846 + 360	11,140
	Altitude (SRTM - NASA)	1	18	5,570
	Geomorphology (IBGE)	10	180	5,570
	Soils (IBGE)	65	1170	5,570
	Phytophysiognomies (IBGE)	52	936	5,570
	Biome boundaries (IBGE)	6	108	5,570
No temporal regularity	Mining areas (ANM)	336	6048	5,570
	Roads (IBGE)	1	18	27,850
	Railways (IBGE)	1	18	27,850
	Waterways or watercourse (IBGE)	2	36	27,850
	Hydroelectric plants (IBGE)	1	18	27,850
	Dams (IBGE)	1	18	27,850
	Conservation unit areas (MMA)	1	18	5,570
	Indigenous lands and Quilombola territories (IBGE)	1	18	5,570
	Zone climates and regional subunits (IBGE)	51	918	5,570
Total		642	11,556	902,224

The column number of attributes is calculated as the *number of classes* × 18 (number of statistics), whereas *number of instances* is the *number of years* × 5,570 (number of municipalities).

**Table 2:** Original data format, resulting geometry, unit and scale/resolution of the thematic layers.

Thematic layers	Original data format	Resulting geometry	Unit of measurement	Scale or spatial resolution
Land use and cover (Mapbiomas v6.0)	raster	polygon	m^2^	30 m
Temperature and precipitation (NCEP/CFSR and CHIRPS)	raster	point	Kelvin, mm	5 km
Population count (SEDAC - NASA)	raster	point	quantity	1 km
Population density (SEDAC - NASA)	raster	point	quantity/km^2^	1 km
Climate normals for temperature and precipitation (Worldclim)	raster	point	°C × 10, mm	1 km
Altitude (SRTM - NASA)	raster	point	m	30 m
Geomorphology (IBGE)	vector	polygon	m^2^	1:5,000,000
Soils (IBGE)	vector	polygon	m^2^	1:5,000,000
Phytophysiognomies (IBGE)	vector	polygon	m^2^	1:5,000,000
Biome boundaries (IBGE)	vector	polygon	m^2^	1:5,000,000
Mining areas (ANM)	vector	polygon	m^2^	1:1,000,000
Roads (IBGE)	vector	line	m	1:250,000
Railways (IBGE)	vector	line	m	1:250,000
Waterways or watercourse (IBGE)	vector	line	m	1:250,000
Hydroelectric plants (IBGE)	vector	point	quantity	1:250,000
Dams (IBGE)	vector	line	m	1:250,000
Conservation unit areas (MMA)	vector	polygon	m^2^	1:100,000
Indigenous lands and Quilombola territories (IBGE)	vector	polygon	m^2^	1:250,000
Zone climates and regional subunits (IBGE)	vector	polygon	m^2^	1:5,000,000

**Table 3:** Statistics calculated for the features/variables in the scope of the municipalities.

Statistics	Description
count	Quantity of features/geometries for each class or variable in the thematic layers contained in each municipality
sum	Sum of the areas, lengths, or values of each class or variable in the thematic layers contained in each municipality
mean	Mean area, length, or value for each class or variable in the thematic layers contained in each municipality
sd	Standard deviation of the areas, lengths, or values for each class or variable in the thematic layers contained in each municipality
min	Minimum area, length, or value for each class or variable in the thematic layers contained in each municipality
max	Maximum area, length, or value for each class or variable in the thematic layers contained in each municipality
25^*th*^ percentile (perc25)	First quartile of the areas, lengths, or values of each class or variable in the thematic layers contained in each municipality
50^*th*^ percentile (perc50)	Median of the areas, lengths, or values of each class or variable in the thematic layers contained in each municipality
75^*th*^ percentile (perc75)	Third quartile of the areas, lengths, or values of each class or variable in the thematic layers contained in each municipality
*_normalized* suffix	This means that the statistic preceding the suffix was divided by the municipality’s area in m^2^

The annual thematic layers for land use and land cover include 25 thematic classes from 1985 to 2020 for the entire Brazilian territory with spatial resolution of 30 m. (Except for the Fernando de Noronha archipelago, municipality geocode 260545, for which there is no land user/cover data due to the absence of historical series Landsat satellite images for that region.) These layers were produced and made available by the online platform MapBiomas^15^, collection 6.0. Annual land use and land cover maps were produced via automatic classification processes applied to Landsat satellite images^16^. The MapBiomas Project is a multi-institutional initiative coordinated by the Greenhouse Gas Emissions Estimation System (SEEG) from the Climate Observatory’s and consists of a collaborative network of cocreators including nongovernmental organizations (NGOs), universities, and companies. The objective is to produce annual land cover and land use maps of Brazil from 1985 to the present.

The annual temperature and precipitation layers include 19 different types of data from 1981 to 2020 for the entire land surface, with spatial resolution of 5 km (0.05°). These fields were derived from two different observational gridded datasets, one for precipitation and another for temperature. The observed precipitation came from the Climate Hazards Group Infrared Precipitation with Stations data (CHIRPS)^17^, with a daily temporal resolution and a spatial resolution of approximately 5 km (0.05°). The observed temperature drawn from the NCEP Climate Forecast System Reanalysis (NCEP/CFSR)^18^ at a 6-hour temporal resolution and a spatial resolution of approximately 50 km (0.5°). The NCEP/CFSR gridded dataset was spatially downscaled to a higher spatial resolution of 5 km (0.05°) using bilinear interpolation in order to have the same spatial resolution as CHIRPS. (As with land use and land cover, there is no temperature/precipitation data for the Fernando de Noronha archipelago (geocode 260545).)

The quinquennial layers for Population Count and Population Density were obtained from the Socioeconomic Data and Applications Center (SEDAC)^19^ through NASA’s Earth Observing System Data and Information System (EOSDIS), and is hosted by the Center for International Earth Science Information Network (CIESIN) at Columbia University. This dataset estimates the population count for the years 2000, 2005, 2010, 2015 and 2020, based on national censuses and population records, and is available in raster graphics with spatial resolution of 1 km. The official population demographics data from IBGE census is not used because it is available only as a tabular data aggregate count per census sector or municipality and therefore cannot yield meaningful descriptive statistics.

Atemporal data include the following themes: Climatological Normals for Temperature; Altitude; Geomorphology; Soils; Phytophysiognomies; and Biome boundaries. Climatological Normals for Temperature came from Worldclim^20^ and correspond to observational data, representative of 1950 to 2000, which were interpolated to a resolution of 1 km. These temperature values are in degree Celsius, but for historical reasons they are scaled by a factor of 10. The used mean, minimum and maximum values of temperature include information from different remote sensors onboard the MODIS and NOAA satellites which operate to jointly capture surface temperature and air humidity values. Besides the annual temperature data, we also included climatological normal data because they provide monthly mean values for temperature. These values complement the annual information (considerably influenced by climate events like El Niño and La Niña) and serve as an important reference on seasonal temperature variation patterns, a factor that directly influences the reproduction and survival dynamics of species such as vectors. The altitude data came from NASA’s Shuttle Radar Topography Mission digital elevation model (SRTM) 1 ArcSecond Global, conceived to provide consistent high-quality near-global elevation data^21^. The original data are radar images with spatial resolution of 30 m, version 3, reprocessed to fix inconsistencies and fill missing data (“voids”). The other themes–Geomorphology, Soils, Phytophysiognomies, and Biome boundaries–were obtained from IBGE^22^. These provides regional details, and were constructed from interpretation of satellite images and various field studies throughout Brazil beginning in 1990^23^.

The layers without temporal regularity include: Mining Areas; Roads; Railways; Waterways or watercourses; Hydroelectric Plants; Dams; Conservation Units; Indigenous Lands; and Zone Climates and Regional Subunits. The Mining Areas layer has 336 classes, representing the different types of minerals explored in Brazil’s territory, provided by the Brazilian National Mining Agency (ANM). The boundaries of Conservation Units were provided by the Brazilian Ministry of Environment (MMA). The other layers are single classes of Roads, Railways, Waterways/watercourses, Hydroelectric Plants, Dams, obtained from the Continuous Cartographic Bases^24^ and Indigenous lands and Quilombola territories^25^, all this datasets from IBGE. The roads category comprises all its available classifications, covering data from subcategories such as highways and dirt roads. The same unification was adopted for the railways and waterways categories. The layer on Zone Climates and Regional Subunits represents the different climate zones in Brazil’s territory, grouped by temperature and humidity. This layer also identifies the climate types, characterized by shades and hues: tropical, subtropical, mild mesothermal, and median mesothermal^26^.

Considering the heterogeneity of the data sources and the structural particularities of the thematic layers acquired, it was essential to conduct a pre-processing and structuring stage with the datasets in order to proceed with the calculation of the descriptive statistics. All the raw data, whose total size amounted to 195 GB, were pre-processed in QGIS v3.10^27^. This stage required standardizing the geospatial data’s cartographic characteristics, correcting topological errors, eliminating duplicate information, and uniformizing the attribute tables. The data were generally organized in two major groups: vector data and matrix data (raster).

To be able to process the Land Use and Land Cover features at the original 30 m spatial resolution, we had first to break down each annual raster (1985 to 2020) into 5,569 smaller raster pieces, one for each municipality, by using the gdalwarp tool from the Geospatial Data Abstraction Library (GDAL). Next, we converted all the resulting rasters to vector format (geopackage) via the script gdal_polygonize.py, also from GDAL. The conversion was necessary because the vector format (geopackage) allowed the calculation of the polygons’ statistics for all the Land Use and Land Cover features, which is not possible with the raster format with the techniques and functions used (described in the Code availability section). All that pre-processing took about 600 hours running in parallel on an Intel Core i7 computer with 8 physical CPU cores and 64 GB of RAM.

The data on Temperature, Precipitation, Population Count/Density, Altitude, and Climatological Normals, also provided in matrix format, were converted to point geometry, since they are inherently points but which had been interpolated by their sources before making them available. The conversion of Altitude from raster to vector was the most computationally demanding operation due to the need to process 10.6 billion points (spread across 821 tiles of 3601 × 3601 points each) at the resolution of 30 m. It took about one month of uninterrupted parallel processing on a 20-core Intel Xeon E5-2690 machine with 128 GB of RAM.

For the vector data, it was first necessary to homogenize the cartographic references using South America Albers Equal Area Conic (EPSG:102033) for data requiring calculation of areas (polygons), South America Equidistant Conic (EPSG:102032) for data requiring calculation of distances (lines), and SIRGAS 2000 Geodetic Reference (EPSG:4674) for data with restricted localization (points)^28^. It was also necessary to correct some topological errors in the vector data regarding the line and polygon geometries, which are artifacts introduced during the data construction/vectorization stage. The vector data correspond to the following themes: Geomorphology; Soils; Phytophysiognomies; Biome Boundaries; Mining Areas; Roads; Railways; Waterways or watercourses; Hydroelectric Plants; Dams; Conservation Units; Indigenous lands and Quilombola territories; Zone Climates and Regional Subunits.

For the statistical description of the municipalities’ socioenvironmental characteristics, we calculated the measures of central tendency such as mean and median, and measures of dispersion such as maximum and minimum values, standard deviation, and percentiles. For each descriptive statistic we also calculated a corresponding normalized statistic, simply dividing the original statistics value by the municipality’s area. The values were normalized due to the wide variation in the territorial area of Brazil’s municipalities. For example, Altamira, in the state of Pará, is Brazil’s largest municipality, with an area of 159,533 km^2^, while Santa Cruz de Minas, in the state of Minas Gerais, is the smallest one, with only 3,565 km^2^ ^29^. This wide territorial variability might otherwise skew the modeling towards the identification of distorted correlations, such as the identification of relations between higher proportions of natural or anthropic features and higher concentration of cases, which is merely due to the municipality’s larger territorial dimensions.

Based on structuring of the graphic, we executed a spatial data intersection with the municipal boundaries by means of different routines from PostGIS^30^, an extension that adds spatial and geographic objects to the PostgreSQL object-relational database.

### Calculation of the descriptive statistics

The meaning of the statistics described in Table 3 actually depends on both *feature’s geometry* and *unit of measurement*, which are reported in Table 2 for each thematic layer.

For *polygons*, such as conservation units, the area of each unit is computed in square meters and the set of all conservation units’ areas in the municipality forms the statistical population upon which the descriptive statistics will be calculated for that municipality. This means that the *minimum* statistic will refer to the smallest area among the conservation units in the municipality, the *mean* statistic to the average area, the *count* statistic will refer to the number of conservation units in the municipality, and so forth. Analogously, when the feature type is *line*, e.g. roads, the set of all road stretches’ lengths (in meters) is the statistical population.

The procedure differs a bit for *point* features, such as altitude and temperature. In this case, except for the *count* statistic (which refers to the number of points in the municipality), the actual value at each feature point is taken; for instance, the altitude and temperature at a given location. Differently from the *polygons* and *line* cases, the associated unit cannot be predefined (in square meters or meters), and it will depend on the actual unit of the underlying layer–for altitude it is meters, but for temperature it could be either Celsius or Kelvin. Some point-type features, such as hydroelectric plants, do not have a unit *per se*, i.e. they merely refer to a *quantity*. Once the set of all point-type feature values are taken, we have a statistical population of values and the calculation of the statistics proceeds exactly as described with the other two feature types.

For each descriptive statistic, there is a corresponding normalized one which is calculated by dividing the statistic by the municipality’s area (in m^2^). Those normalized statistics complement the set of descriptive information and provide the notion of proportion or density. As an example, the statistic *sum_normalized* corresponds to the percentage of occupation of a given polygon-type thematic layer in the municipality, or an estimation of density for line-type layers such as roads.

## Data Records

The complete collection comprises 22 datasets in CSV format, with 21 datasets containing each the descriptive statistics of the variables relative to a thematic layer for each of the 5,570 municipalities, and an additional dataset that contains municipalities’ information (name, state, region and area in m^2^). Overall there are 642 sets of descriptive statistics distributed among the datasets, totaling 11,556 statistics attributes. The dataset collection along with the data dictionary as a PDF document describing the 642 variables are available for downloading at Open Science Framework (OSF)^31^. The dataset collection has a size of roughly 2.6 GB and the data dictionary about 3 MB. Each CSV dataset has a header and the records are organized as follows:

1. The first field is named geocode and corresponds to the municipality’s geocode;
2. The second field, datetime, is the approximate year (YYYY format) on which the thematic layer–not the statistics–was gathered;
3. The remaining fields are the sets of 18 descriptive statistics for all variables, i.e. the statistics of the municipality’s socioenvironmental characteristics; those fields are labeled according to the grammar <variable>-<statistic>. Examples include *Altitude-max*, which corresponds to the maximum Altitude value, and *Urban_infrastructure-perc75_normalized*, the normalized (by municipality’s area) 75^*th*^ percentile of Urban infrastructure areas (Tables 4–6).

**Table 4:** Values of descriptive statistics calculated in PostgreSQL/PostGIS for the Urban Infrastructure class in the municipality of Rio de Janeiro (areas in m^2^).

geocode	datetime	Urban_infrastructure-count	Urban_infrastructure-sum	Urban_infrastructure-mean	Urban_infrastructure-sd	Urban_infrastructure-min	Urban_infrastructure-max
330455	2020	344	667214841.91	1939578.03	32714160.03	358.29	607070012.49

The *count* statistic refers to the number of urban areas.

**Table 5:** Values of descriptive statistics calculated in PostGIS/PostgreSQL for the Roads class in the municipality of Rio de Janeiro (lengths in meters).

geocode	datetime	Roads-count	Roads-sum	Roads-mean	Roads-sd	Roads-min	Roads-max
330455	2013	118	333061.81	2822.56	2876.17	16.65	12426.91

The *count* statistic refers to the number of roads.

**Table 6:** Values of descriptive statistics calculated in PostGIS/PostgreSQL for the Altitude variable in the municipality of Rio de Janeiro (in meters).

geocode	datetime	Altitude-count	Altitude-sum	Altitude-mean	Altitude-sd	Altitude-min	Altitude-max
330455	2000	1369182	119077300	86.97	147.50	−28	1014

The *count* statistic refers to the number of altitude data points.

Regarding the temporality, in Table 7 the reader can find a list of all the years for which there are calculated statistics for each thematic layer.

**Table 7:** List of available years for each thematic layer.

Thematic layers	Available years
Land use and cover (Mapbiomas v6.0)	1985 to 2020
Temperature and precipitation (NCEP/CFSR and CHIRPS)	1981 to 2020
Population count (SEDAC - NASA)	2000, 2005, 2010, 2015, 2020
Population density (SEDAC - NASA)	
Climate normals for temperature and precipitation (Worldclim)	Climate normals from 1950 to 2000
Altitude (SRTM - NASA)	2000
Geomorphology (IBGE)	2006
Soils (IBGE)	2006
Phytophysiognomies (IBGE)	2004
Biome boundaries (IBGE)	2006
Mining areas (ANM)	2021
Roads (IBGE)	2013, 2015, 2017, 2019, 2021
Railways (IBGE)	
Waterways or watercourses (IBGE)	
Hydroelectric plants (IBGE)	
Dams (IBGE)	
Conservation unit areas (MMA)	2020
Indigenous lands and Quilombola territories (IBGE)	2019
Zone climates and regional subunits (IBGE)	2002

## Technical Validation

**Fig. 5:**
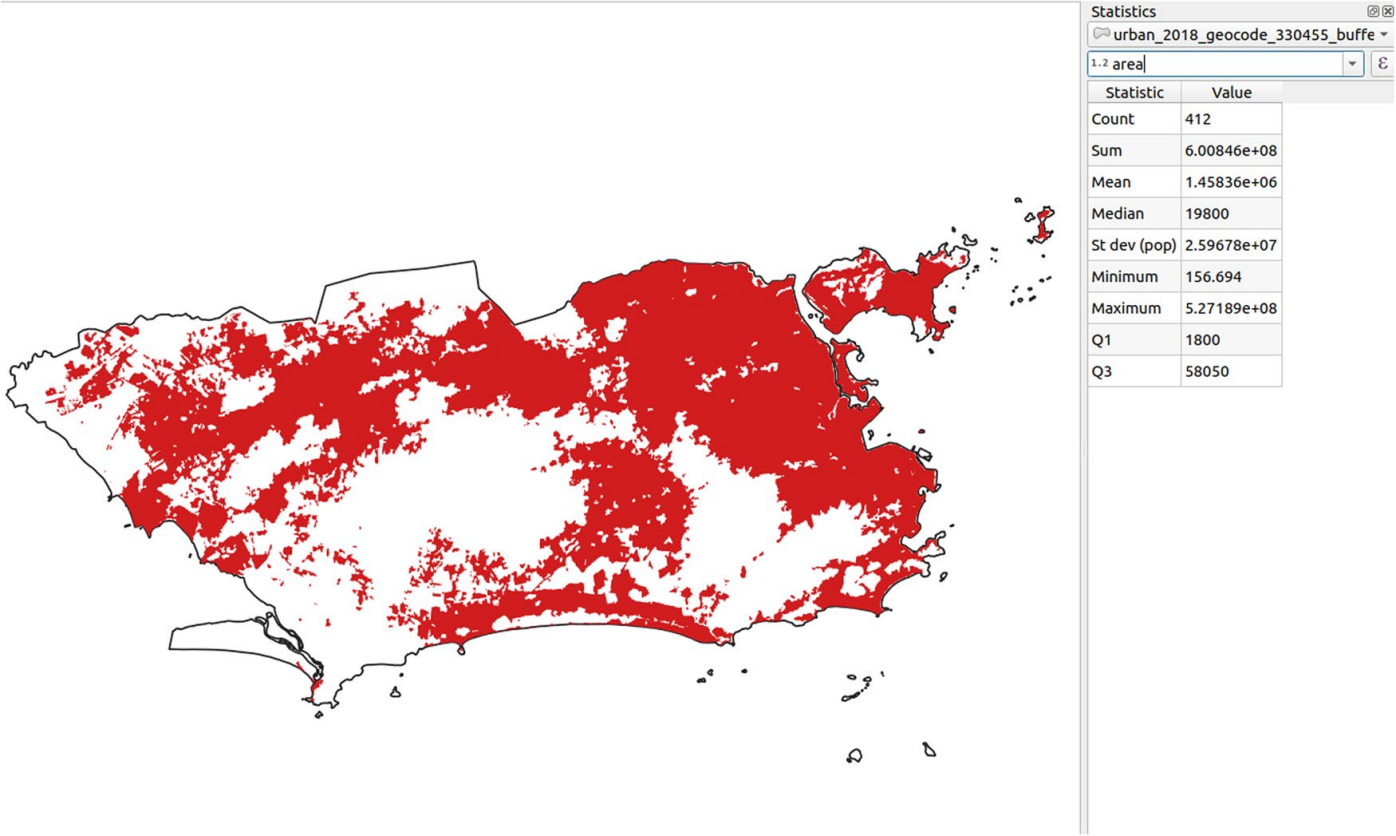
Values of descriptive statistics calculated in QGIS for the Urban Infrastructure class in the municipality of Rio de Janeiro (areas in m^2^). The *count* statistic refers to the number of urban areas.

**Fig. 6:**
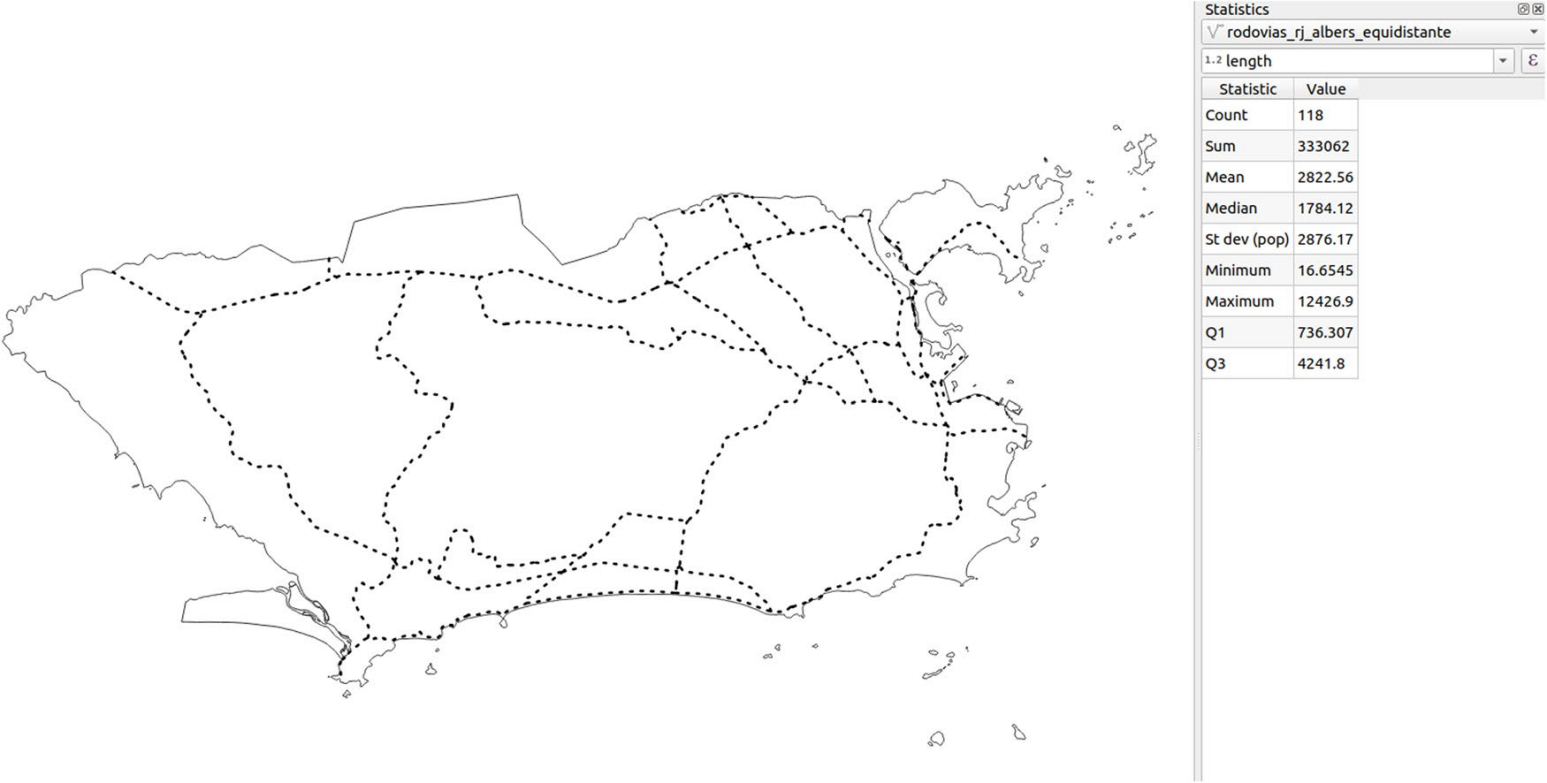
Values of descriptive statistics calculated in QGIS for the Road class in the municipality of Rio de Janeiro (lengths in meters). The *count* statistic refers to the number of roads.

**Fig. 7:**
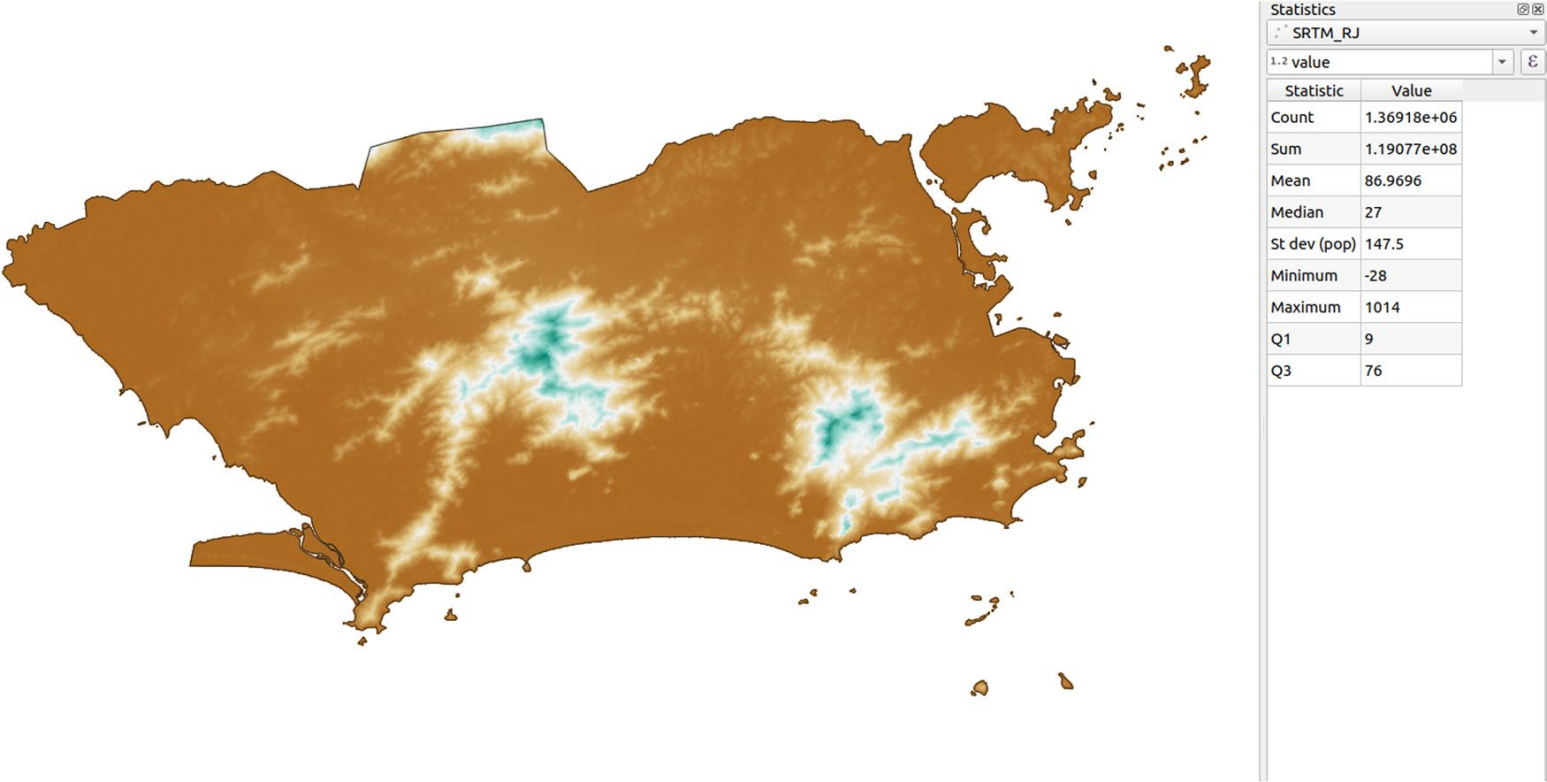
Values of descriptive statistics calculated in QGIS for the Altitude variable in the municipality of Rio de Janeiro (in meters). The *count* statistic refers to the number of altitude data points. The points colored in brown have lower altitudes, in white intermediate altitudes, and in green the highest altitudes.

The proposed datasets were validated by comparison of the final descriptive statistics calculated by two methods from different software platforms: one was using the PostGIS scripts illustrated in the Code availability section, whose the resulting statistics can be seen in Tables 4–6; and the other one using the spatial analysis tools from QGIS v3.10, shown respectively in Figs. 5–7.

Since this was a validation stage, only exemplars of each of polygon, point, and line-type thematic layers of randomly sampled municipalities were used for comparison. For brevity, the tables and figures only describe the statistics for the Rio de Janeiro municipality (geocode 330455) with respect to the following thematic layers: Urban Infrastructure (polygon geometry, in square meters); Roads (line geometry, in meters); and Altitude (point geometry, in meters). First, it was necessary to perform a spatial intersection of each layer with the municipality of Rio de Janeiro using the clip tool, thus extracting the geometries that overlay the municipality’s region. Afterwards, the area and length of each geometry were calculated, respectively for the Urban Infrastructure and Roads. Since Altitude is a point geometry, the statistics were calculated for the values rather than the geometries. After obtaining the values for each geometry in each thematic class/variable, the statistics were verified upon the attributes table, in which it is possible to describe the statistics in numerical-type fields (how many values the field has, as well as the sum, minimum, mean, maximum and standard deviation of those values).

As demonstrated in the validation process, the statistics produced by means of the PostGIS script could also be obtained via tools available in graphical Geographic Information Systems (GIS). However, the scripting streamlined the process, since the SQL queries do not require rendering of the geometric features and also no user interaction is required.

## Usage Notes

The proposed dataset can be used in a myriad of ways in various research fields; in practice, any study that relies on summary statistics of socioenvironmental characteristics of Brazil’s territory (up to the scale of municipalities) over time can take advantage of the dataset. Still, the dataset was originally conceived to be used in ecological and emerging diseases studies whose objective is to identify consistent patterns or systematic relations between socioenvironmental conditions in geographic space and the occurrence of different zoonotic diseases. In particular, we have been using extensively the dataset in the context of data-driven modeling of Sylvatic Yellow Fever occurrences by means of machine-learning algorithms. In this category of application, a training dataset is assembled by joining the presence of Yellow Fever Virus (YFV) in humans and non human primates with the socioenvironmental statistics dataset, using the municipalities’ geocodes as joining keys.

As a hypothetical example, suppose for instance that there are reported cases of a given zoonotic disease on the municipalities identified by the geocodes *X* and *Y*. Suppose also that municipality *Z* is known not to be susceptible to the disease (according to some evidences). Considering only the mean altitude for simplicity (out of thousands of attributes), imagine that their values are, respectively for *X*, *Y* and *Z*: 415.3 m, 560.7 m and 124.0 m. The resulting training dataset ready for learning a binary classification model would be like the one described in Table 8. A machine-learning model for this hypothetical example would possibly separate the positive from the negative cases by using the altitude of 306 m as the threshold, thus suggesting a hypothesis of how the disease is associated with the socioenvironmental characteristics. If we were to predict the likelihood of disease occurrence of a municipality with mean altitude of 350 m, this municipality would be classified as *positive* according to this imaginary model. By applying trained models to all the Brazilian municipalities we would end up with a susceptibility map, which would be helpful to identify priority areas for health surveillance, control and prevention actions, such as vaccination.

**Table 8:** Illustrative example of an assembled training dataset.

Mean altitude (m)	occurrence
415.3	yes
560.7	yes
124.0	no

It is worth noticing that, since the proposed dataset consists of thousands of attributes–which typically outnumbers by a large margin the amount of disease cases–a pre-processing step for reducing the attributes dimension is required in order to make the learning problem well posed^32^.

## Data Availability

Initially, all the thematic layers and municipal boundaries were imported to a database created in the database management system PostgreSQL/PostGIS via the ogr2ogr, a command line tool^33^. Scripts were then developed to calculate the statistics. These scripts used regular SQL queries and the ST_AREA and ST_INTERSECTION spatial functions from PostGIS. We also used the aggs_for_arrays plugin, version 1.3.2^34^, which is an extension that offers various functions for optimized computation of different statistics on array of numbers. The calculation of the descriptive statistics for the 642 thematic classes/variables took more than three hundred hours of parallel processing on an Intel Core i7 desktop workstation equipped with 8 physical CPU cores and 64 GB of RAM. The following is an example of a script for calculating the maximum forest area statistics per municipality. This same script were used for all of them: By using similar PostGIS scripts with slightly different statistics functions we were able to calculate for all the 5,570 Brazilian municipalities the following statistics: count, minimum, maximum, mean, sum, standard deviation, 25^*th*^ percentile, 50^*th*^ percentile (median), and 75^*th*^ percentile, and the normalized counterparts as well (Table 3). All the statistics were stored in temporary tables, which were aggregated via the INNER JOIN function (using the geocode of each municipality as the junction field) in a final table. At the end of the execution, the per-municipality statistics for each layer were integrated and structured as a collection of datasets containing 11,556 attributes that represent the temporal and spatial plurality of the main socioenvironmental characteristics of the Brazilian municipalities.
